# Non-autoimmune hyperthyroidism caused by thyroid-stimulating hormone receptor germline mutations - 2012 European Thyroid Association Guidelines

**DOI:** 10.1186/1756-6614-6-S2-A45

**Published:** 2013-04-05

**Authors:** R Paschke, M Niedziela, B Vaidya, L Persani, B Rapoport, J Leclere

**Affiliations:** 1 Department of Endocrinology and Nephrology, Leipzig University, Leipzig, Germany; 2 Department of Pediatric Endocrinology and Rheumatology, Poznan University of Medical Sciences, Poznan, Poland; 3 Department of Endocrinology, Royal Devon and Exeter Hospital, Peninsula Medical School, Exeter, UK; 4 Department of Clinical Sciences, and Community Health, University of Milan, and Istituto Auxologico Italiano, Milan, Italy; 5 Autoimmune Disease Unit, Cedars-Sinai Research Institute and School of Medicine, University of California, Los Angeles, CA, USA; 6 Centre Hospitalier Universitaire de Nancy, Nancy, France

## 

All cases of familial thyrotoxicosis with absence of evidence of autoimmunity and all children with persistent isolated neonatal hyperthyroidism should be evaluated for familial non-autoimmune autosomal dominant hyperthyroidism (FNAH) or persistent sporadic non-autoimmune hyperthyroidism (PSNAH). First, all index patients should be analysed for the presence/absence of a thyroid-stimulating hormone (TSH) receptor (*TSHR*) germline mutation, and if they display a *TSHR* germline mutation, all other family members including asymptomatic and euthyroid family members should also be analysed. A functional characterization of all new *TSHR* mutations is necessary. Appropriate ablative therapy is recommended to avoid relapses of hyperthyroidism and its consequences, especially in children. Therefore, in children the diagnosis of FNAH or PSNAH needs to be established as early as possible in the presence of the clinical hallmarks of the disease.
